# Deep Q-Learning for Gastrointestinal Disease Detection and Classification

**DOI:** 10.3390/bioengineering12111184

**Published:** 2025-10-30

**Authors:** Aini Saba, Javaria Amin, Muhammad Umair Ali

**Affiliations:** 1 Department of Computer Science, University of Wah, Wah Cantt 47040, Pakistan; aini786saba@gmail.com (A.S.); javeria.amin@f.rwu.edu.pk (J.A.); 2 Department of Computer Science, Rawalpindi Women University, Rawalpindi 43600, Pakistan; 3 Department of Artificial Intelligence and Robotics, Sejong University, Seoul 05006, Republic of Korea

**Keywords:** attention U-Net, CNN, classification, medical imaging, Q-learning, stomach ulcers

## Abstract

Stomach ulcers, a common type of gastrointestinal (GI) disease, pose serious health risks if not diagnosed and treated at an early stage. Therefore, in this research, a method is proposed based on two deep learning models for classification and segmentation. The classification model is based on Convolutional Neural Networks (CNN) and incorporates Q-learning to achieve learning stability and decision accuracy through reinforcement-based feedback. In this model, input images are passed through a custom CNN model comprising seven layers, including convolutional, ReLU, max pooling, flattening, and fully connected layers, for feature extraction. Furthermore, the agent selects an action (class) for each input and receives a +1 reward for a correct prediction and −1 for an incorrect one. The Q-table stores a mapping between image features (states) and class predictions (actions), and is updated at each step based on the reward using the Q-learning update rule. This process runs over 1000 episodes and utilizes Q-learning parameters (α = 0.1, γ = 0.6, ϵ = 0.1) to help the agent learn an optimal classification strategy. After training, the agent is evaluated on the test data using only its learned policy. The classified ulcer images are passed to the proposed attention-based U-Net model to segment the lesion regions. The model contains an encoder, a decoder, and attention layers. The encoder block extracts features through pooling and convolution layers, while the decoder block up-samples the features and reconstructs the segmentation map. Similarly, the attention block is used to highlight the important features obtained from the encoder block before passing them to the decoder block, helping the model focus on relevant spatial information. The model is trained using the selected hyperparameters, including an 8-batch size, the Adam optimizer, and 50 epochs. The performance of the models is evaluated on Kvasir, Nerthus, CVC-ClinicDB, and a private POF dataset. The classification framework provides 99.08% accuracy on Kvasir and 100% accuracy on Nerthus. In contrast, the segmentation framework yields 98.09% accuracy on Kvasir, 99.77% accuracy on Nerthus, 98.49% accuracy on CVC-ClinicDB, and 99.13% accuracy on the private dataset. The achieved results are superior to those of previous methods published in this domain.

## 1. Introduction

One of the most prevalent diseases that results in a significant number of deaths annually is gastrointestinal disease. As they generate substantial health problems for a large number of people, gastrointestinal (GI) ailments are among the worldwide illnesses that require immediate attention. These conditions include Ulcerative Colitis (UC) and Crohn’s Disease (CD), which are types of inflammatory bowel disease, colorectal cancer, and several forms of gastritis [1]. Since ongoing monitoring, therapy, and operations typically cost a significant amount of money, their detrimental effects manifest as a discernible decline in patients’ standard of living and a substantial rise in healthcare expenses [2]. Furthermore, the increase in colorectal cancer and other GI disorders has coincided with an exponential rise in other types of cancer. Due to the higher risk of cancer, GI disorders have become more common, requiring early and prompt identification and categorization of GI conditions to enhance patient care [3]. Although endoscopy scans are now sufficient for assessing gastrointestinal disorders, they need to be interpreted carefully and may not always provide a reliable diagnosis [4]. At varying degrees of consistency and objectivity, these evaluation procedures have demonstrated successful outcomes in the consistent and accurate categorization of several gastrointestinal disorders, which were not previously achievable with conventional approaches [5]. The most common disease associated with the stomach is colorectal cancer. It has an impact on both men and women. Polyps, ulcers, and bleeding are the three major disorders that lead to colorectal cancer. An ulcer is a challenging, inflamed lesion. Gastric ulcers are also known as stomach ulcers [6]. When it comes to prevalence, colorectal cancer ranks third in males and ranks second in women. Since polyps are symptoms of colorectal cancer, it is significant to find and remove them at an early stage. Polyps are seen in about half of the people who receive a colonoscopy test at age fifty, and their prevalence increases with age. Unusual cell formations known as polyps arise from the mucous membrane lining the intestinal tract. These growths can occasionally be malignant. The most effective method for identifying and evaluating these polyps is a colonoscopy, which includes a biopsy and removal of the polyp. The prognosis for colorectal cancer is greatly impacted by early disease identification. According to the WHO, Colon cancer is one of the biggest reasons for death from cancer globally and the third-most prevalent cancer overall, making up over 10% of the worldwide incidence of cancer. Multiple factors may increase the chance of colorectal cancer such as age (a large quantity of colorectal cancer diagnoses occur in those over 50, and the risk increases with age), family history (colorectal cancer occurring in close relatives, as well as some genetic illnesses including a history of stomach ulcers, along with genetic disorders like Lynch syndrome and FAP, can contribute to an increased risk of gastrointestinal complications or certain types of polyps), and lifestyle (choices about an unhealthy way of living might increase risk factors). A few examples are passive obesity, smoking, excessive alcohol consumption, and diets that are high in junk food and low in fruits and vegetables [7]. In its initial phases, colorectal cancer shows no signs at all. Routine testing is necessary to identify the illness early and begin treatment. Some common symptoms of colorectal cancer are as follows: Modifications in eating routines, such as constipation, diarrhea, or stool narrowing. Stool (bowel bleed), which can be black and tarry or bright crimson, persistent bloating, discomfort, or cramping in the abdomen. Sudden, unexpected weight reduction that occurs without conscious effort. Constantly being exhausted and deficient in energy, even after obtaining sufficient sleep, and lack of iron hemoglobin brought on by continuous bleeding, resulting in weakness, paleness, and exhaustion. Physical examinations, scanning (such as abdominal ultrasounds, CT, and MRI), colonoscopy or sigmoidoscopy testing, and other modalities are used in the diagnosis of colorectal cancer. Tissue sample examination for the histopathology tests and molecular screening for detecting DNA changes or biomarkers that determine the best course of medication are all diagnostic techniques for colorectal cancer [8]. However, the use of ML may facilitate the diagnosis of intestinal and digestive issues. CNNs and other advanced machine learning algorithms primarily handle the problems of creating training data and identifying small features in external images. These methods need minimal to no human intervention and enhance the clinical information’s impartiality, uniformity, and correctness. Artificial intelligence (AI) algorithms have been used to identify several GI illness classifications from endoscopy images, leading to a quicker, more precise, and non-invasive diagnosis [9]. Reducing incorrect diagnoses by up to 60 times, facilitating initial diagnosis and early therapy application, lowering costs, and achieving better results for patients are just a few of the numerous benefits of ML models [10]. AI methods, such as ML and DL, have generally changed to become more essential for identifying GI disorders, even though algorithms using AI are very beneficial for medical research. To enable accurate results in diagnosing GI disorders, ML methods like CNN-based techniques have been used on endoscopic, histological, and capsule-based endoscopic visuals [11]. Large amounts of data may be examined and subsequently diagnosed more effectively and reliably with the use of such algorithms [12]. Furthermore, ML models enable therapists to evaluate activities more accurately according to their severity, making decisions more quickly and using time and resources more efficiently [13]. Additionally, supervised learning has advanced in some supervised and transfer learning approaches, utilizing AI algorithms. In the future, machine learning (ML) is going to be capable of producing more precise and comprehensive predictions and contribute substantially to the diagnosis of stomach ulcers using endoscopic scans because of continuing efforts to incorporate data from multiple sources, decrease prediction bias, and improve the comprehension of models by utilizing Explainable AI (XAI) [14].

The core contribution steps are as follows:

1. Proposed a two-phase system integrating Q-learning-enhanced CNN for classification and attention-based U-Net for segmentation of gastrointestinal ulcer images.
2. Developed a custom CNN model with seven layers (convolutional, ReLU, max pooling, flatten, and fully connected layer) integrated with Q-learning for stable and accurate classification, where a Q-table maps image features to actions based on reward signals.
3. Designed an attention-based U-Net with encoder, decoder, and attention blocks to accurately segment lesion regions by emphasizing spatially important features extracted from the encoder.
4. The proposed model was evaluated on both publicly available datasets and real patient data to ensure a comprehensive performance assessment.

The paper consists of five sections. Related work is covered in Section 2, the model we suggest is thoroughly explained in Section 3, the model’s outcomes are examined in Section 4, and a conclusion is given in Section 5.

## 2. Related Work

There are several ways to classify gastrointestinal disease (GD), and this section discusses some of the more current classifications [14,15,16,17,18,19,20]. A deep learning approach combining MNETGIDD and MobileNetV2 was developed for gastrointestinal disease detection, and it was evaluated on the GestroLab dataset, achieving a test accuracy of 97% [21]. The Deep Hexa model and UNet were utilized for gastrointestinal multiclass classification, achieving a 99% accuracy rate on two datasets, Kvasir and KID [22]. A GINet and CNN model was designed, resulting in an impressive 99% accuracy for gastrointestinal disease detection on the Kvasir Capsule dataset [23]. Gastrointestinal disease detection runs a vibrant field of study. Models such as CRC Fusion AICADx and CNN-LSTM have been deployed for GD, achieving an accuracy of 98% on the CKHK-22 dataset [24]. CFFormer was employed using Kvasir-SEG, CVC-Clinic, and Brain MRI datasets, with preprocessing techniques such as modified thresholding and histogram equalization [25]. A ColonSegNet DL technique, using Adam’s optimizer, yielded better accuracy on three datasets: Kvasir, Kavasir-SEG, and Mask Extraction [26]. The DUNSDL model, when combined with different classifiers, achieved an accuracy of 97% in GD classification on the CVC-Clinic DB dataset [27]. SHAP, SoTA, GreedySoup, GA, and DenseNet121, which analyzed volumetric and grid-based features, achieved 80% accuracy in classification XAI after analyzing two datasets, GradCAM and GastroVision [28]. Y. Oukdach et al. [29] introduced the Leveraging Knowledge Distillation (KD) model along with CNN and ViT, which facilitates early-stage gastrointestinal disease detection with a 96% success rate on Kvasir and KID datasets. The LSTM, DarkNet-19, ResNet-50, and DenseNet-201, when pre-trained and paired with different classifiers, classified GD with an accuracy of 93% on the HyperKvasir [30]. Multiple models, including EfficientNetB0, MobileNetV2, and ResNet50, were utilized for the classification of gastrointestinal disease detection, achieving 98% accuracy on two datasets: Kvasir and WCE, curated by [31]. Employing a Self-Attention and Bayesian optimization model along with a Shallow Wide Neural Network for GD classification, and testing on the KvasirV1 and KvasirV2 datasets, yielded a staggering 99.60% and 95% accuracy respectively [32]. The Kvasir-SEG, CVC-Clinic DB, and Saga University datasets were utilized to evaluate a Dyadic Wavelet Packet Transform with SHAP [33]. The CMNet and AAM models were used for GD detection, achieving 95% accuracy on two datasets: Kvasir-SEG and CVC-ClinicDB [34]. TFCNet, TAM, TFEM, and RPSA were developed to cater to the morphological changes observed in GD, achieving an mAP@0.5 of 95% on the CVC-Clinical DB dataset [35]. To tackle the multiclass challenge in GD, DRKNET-53 and Xception V3 were tested on the Kvasir dataset with an accuracy rate of 98% [15]. Lastly, an InceptionV3 CNN model, along with SVM, achieved 93% accuracy on the Kvasir dataset [36].

## 3. Proposed Methodology

**Figure 1:**
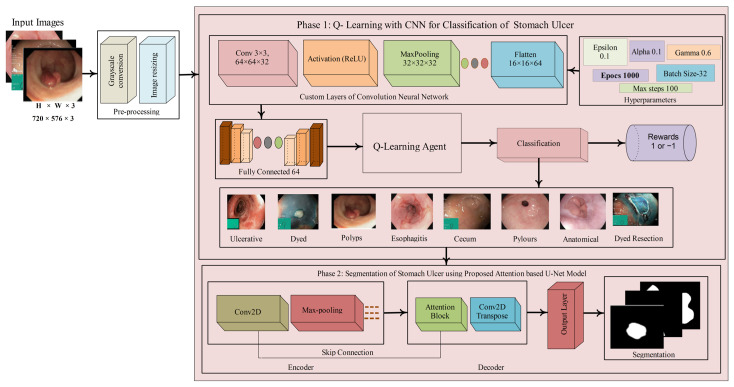
Steps of the proposed method for classification and segmentation models.

The comprehensive review of existing research on stomach ulcer (SU) detection, as discussed in the previous section, highlights ongoing challenges and the potential for further advancement in this field. The presence of image artifacts, such as low contrast and noise, often hampers accurate detection. To address these challenges, a method is proposed that comprises two primary stages: classification and segmentation. The classification model was trained from scratch using selected hyperparameters as given in Figure 1.

In Figure 1, SU images are classified using the proposed Q-Learning with CNN model.

### 3.1. Stomach Ulcer Classification Using the Proposed Q-Learning with CNN Model

In the proposed framework, the CNN extracts high-level spatial features from input images, which are then treated as states in a reinforcement learning environment. The Q-Learning algorithm uses these states to make classification decisions, updating its knowledge through reward signals.

### 3.2. CNN Architecture and Feature Extraction

A CNN’s design typically consists of several convolutional and pooling layers, accompanied by fully connected layers. The input image is passed to a set of 32 3×3 filters by the initial CNN layer. This layer’s behavior is

(1) $$z_{1}=W_{1}\times x+b_{1}$$

In Equation (1), where W_1_ stands for the convolutional filters, b_1_ for the bias term, and x for the input, the Rectified Linear Unit (ReLU) is used.

(2) $$a_{1}={ReLU(z}_{1})=max(0,z_{1})$$

In Equation (2), z_1_ is the input of the activation function, and a_1_ is the output of the activation. The spatial dimensions are reduced by a max pooling procedure following the ReLU activation. In Equations (3) and (4), 64 3 × 3 filters compose the second convolutional layer, which is applied to the first layer’s output:

(3) $$z_{2}=W_{2}\times x+b_{2}$$

(4) $$a_{2}={ReLU(z}_{2})=max(0,z_{2})$$

Max pooling also comes after this. In Equation (5), after being flattened, the output of the last pooling layer is sent to a fully connected layer.

(5) $$f={ReLU(W}_{3}.flatten\left(a_{2}\right)+b_{3})$$

The Q-Learning agent uses the resultant vector f ∈ R64 as its input state. The anticipated reward of acting in-state *s* is estimated by each of the Q-values Q(s,a) that Q-Learning keeps track of. The update rule is as follows:

(6) $$Q(s,a)\leftarrow Q(s,a)+\alpha [r+\gamma \cdot {\max_{a}}^{\prime }Q(s^{\prime },a^{\prime })-Q(s,a)]$$

In Equation (6), r is the immediate reward obtained after taking action in in-state s, $s^{\prime }$ is the new state following the action, and *a* represents the action in the current state, while $a^{\prime }$ is the action taken in a new state. γ is the discount factor, which controls the relevance of future rewards, and α is the learning rate, which determines how much new knowledge overrides the old. The reward *r* for image categorization is −1 for an inaccurate prediction and +1 for a correct one.

In Equation (7), to ensure a balance between exploration (trying new actions) and exploitation (choosing known best actions), an ε-greedy policy is adopted. According to this strategy,

(7) $$a=\left\{\begin{array}{cc} \mathrm{random}\;\mathrm{action}, & \mathrm{if}\;\mathrm{rand}()<\varepsilon \\ \;\;\arg\;\max_{a}\;Q(s,a), & \mathrm{otherwise} \end{array}\right.$$

This allows the agent to occasionally explore new actions, preventing premature convergence to a suboptimal policy.

During the training phase, the CNN extracts features from each input image, which are used as the state s for the Q-learning agent. The agent then selects an action (i.e., a class label) using the ϵ greedy policy, receives a reward r, and updates the Q-table according to the update rule.

In the testing phase, the Q-table is used to predict the class labels for unseen images by selecting the action with the highest Q-value for the given state:

(8) Prediction class=maxaQ(s,a)

This allows the agent to occasionally explore new actions, preventing it from converging prematurely to a suboptimal policy. During the training phase, the CNN extracts features from each input image, which are used as the state *s* for the Q-learning agent. The agent then selects an action (i.e., a class label), updates the Q-table according to the update rule, receives a reward r, and applies the ϵ-greedy policy. In Equation (8), the testing phase, the action with the greatest Q-value for the specified state is chosen to utilize the Q-table to predict the class labels for unseen images.

Performance is evaluated by comparing predicted labels against the ground truth. The flow of data in this combined architecture is as follows. The CNN receives the 64 × 64 input images. Two convolutional layer models with max pooling and ReLU activation compose the CNN. The final output is flattened and processed by a fully connected layer to create a 64-dimensional feature vector. The Q-learning agent uses this vector as its state *s*. The Q-learning agent selects an action (i.e., the predicted class) based on the Q-values. A reward is issued depending on the correctness of the prediction. The Q-table is updated accordingly. The primary metric used for evaluation is classification accuracy, computed as

(9) $$Accuracy=(\frac{Number\;of\;Correct\;Prediction}{Total\;Number\;of\;Predictions})\times 100$$

In Equation (9), a confusion matrix is used to analyze performance across individual classes, providing insight into the specific types of classification errors made by the model. The specified hyperparameters listed in Table 1 are used to train the Q-Learning CNN model from scratch.

**Figure 2:**
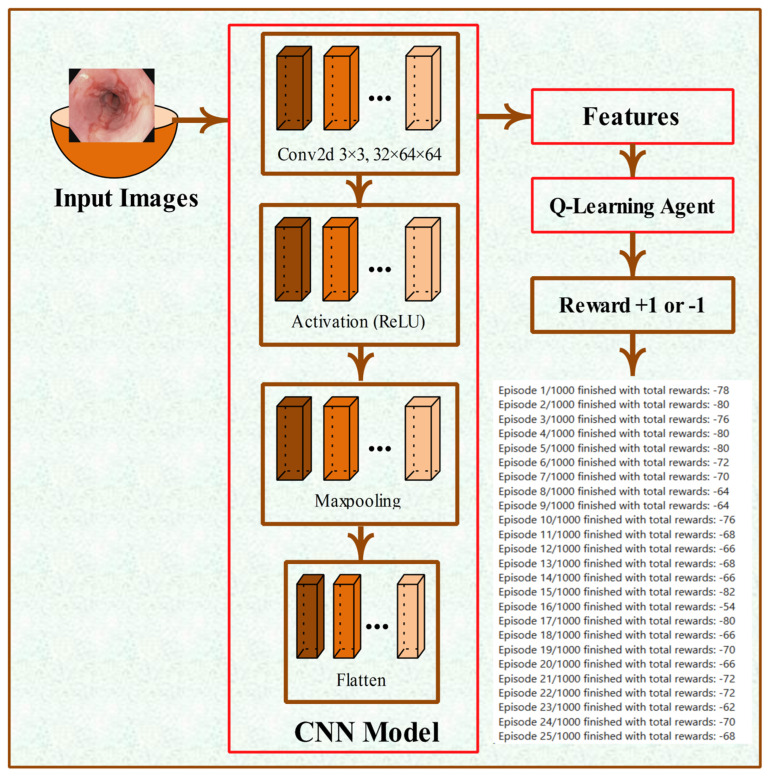
Proposed Layer Architecture of Classification Model.

Table 1 shows the hyperparameters. In this experiment, parameters included α = 0.1, γ = 0.6, ϵ = 0.1, training epochs = 1000, maxsteps = 100, loss = binary cross-entropy, optimizer = AdamW. These parameters provide good results. The proposed layer architecture of the classification model is shown in Figure 2.

### 3.3. Proposed U-Net Model

The function load_data() is responsible for loading the images and their corresponding segmentation masks. Each image and mask is resized to a fixed resolution of 128×128 pixels. The pixel values are standardized to fall between 0 and 1 in order to prepare the data for model training. This normalization is mathematically defined as follows:

(10) $$I^{\prime }=\frac{I}{255}$$

In Equation (10), I represents the original pixel intensity value ranging from 0 to 255, and $I^{\prime }$ denotes the normalized value.

#### 3.3.1. Attention Block

Before starting the concatenation, the attention mechanism is essential for highlighting pertinent spatial characteristics. In medical image segmentation, where small details are crucial, this is essential. Let f represent the attention filters, g the gating signal, and x the map of input features.

In Equations (11) and (12), 1×1 convolutions are applied to project both the input and the gating signal using trainable weight matrices $W_{f}$ and $W_{g}$:

(11) $$f=W_{f}\times x$$

(12) $$g=W_{g}\times g^{\prime }$$

In Equations (13) and (14), these feature maps are resized using bilinear interpolation to match dimensions:

(13) $$g^{\prime }=resize(g,(H_{f},\;W_{f}))$$

(14) $$h^{\prime }=resize(h,(H_{f},\;W_{f}))$$

In Equation (15), Attention coefficients are computed using the sigmoid function:

(15) $$\alpha =\sigma (f+g^{\prime }+h^{\prime })$$

where $\sigma \left(x\right)=\frac{1}{\left(1+e^{-x}\right)}$.

Equation (16) refines the feature map xxx using an attention coefficient α:

(16) $$x^{\prime }=\alpha .x$$

Here, α acts as a weighting factor that highlights important features and reduces the influence of less relevant ones, ensuring that the network focuses on the most informative regions.

#### 3.3.2. Attention U-Net Architecture

The Attention U-Net architecture builds upon the traditional U-Net by incorporating attention gates, especially in the decoder path. The model’s convolutional blocks each have two 3×3 convolution operations and a ReLU, which is written as follows:

(17) $$C_{i}=ReLU(W_{i}\times x+b_{i})$$

In Equation (17), x is the input tensor, bi is the bias, and ${WS}_{i}$ is a learnable filter. A max pooling process is used to carry out down-sampling, as shown in Equation (18):

(18) P=MaxPool(C)

This operation reduces spatial dimensions by a factor of two. For up-sampling, transposed convolutions are used, as shown in Equation (19):

(19) U=Conv2DTranspose(C)

In Equation (19), each up-sampled feature map is passed through an attention block before being concatenated with the corresponding encoder features, ensuring that important information is preserved and irrelevant features are filtered out.

#### 3.3.3. Model Compilation and Training

Binary cross-entropy loss is applied for model training, which is computed using the Adam optimization. The definition of the loss function is

(20) $$L=-\frac{1}{N}\sum_{i=1}^{N}\sum_{c=1}^{C}y_{i,c}log({\hat{y}}_{i,c})$$

In Equation (20), $\hat{y}$ represents the predicted probability for the $i^{th}$ pixel, $y_{i}$ is the ground truth, and *N* is the total number of pixels.

To encourage the model to predict probabilities that are close to the ground truth, this loss function penalizes inaccurate pixel-wise classifications. The overlap between the real and anticipated segmentation masks is measured by the first metric shown in Equation (21):

(21) $$Dice=\frac{2\sum_{i=1}^{N}y_{i}{\hat{y}}_{i}}{\sum_{i=1}^{N}y_{i}+\sum_{i=1}^{N}{\hat{y}}_{i}}$$

Equation (22) shows that IoU measures the intersection over the union of the ground truth and anticipated masks.

(22) $$IOU=\frac{\sum_{i=1}^{N}y_{i}{\hat{y}}_{i}}{\sum_{i=1}^{N}y_{i}\sum_{i=1}^{N}{\hat{y}}_{i}-\sum_{i=1}^{N}y_{i}{\hat{y}}_{i}+\epsilon }$$

where ϵ is a small constant to ensure numerical stability.

Equation (23) calculates the percentage of all pixels that were successfully categorized.

(23) $$Accuracy=\frac{\sum_{i=1}^{N}\left[y_{i}={\hat{y}}_{i}\right]}{N}$$

In Equation (23), $y_{i}$ denotes the ground truth label, ${\hat{y}}_{i}$ the predicted label for the $i^{th}$ pixel, and N the total number of pixels. The term $\left[y_{i}={\hat{y}}_{i}\right]$ evaluates to 1 when prediction matches ground truth, and 0 otherwise. Every indicator offers a distinct perspective on model performance and is crucial for verifying the effectiveness of the segmentation. The Attention U-Net model significantly enhances feature selection in the decoder stages, which is crucial for precise image segmentation tasks, such as those in medical imaging and attention gates that eliminate superfluous features. This architecture is particularly beneficial when dealing with complex segmentation scenarios where identifying small but critical regions is essential.

**Figure 3:**
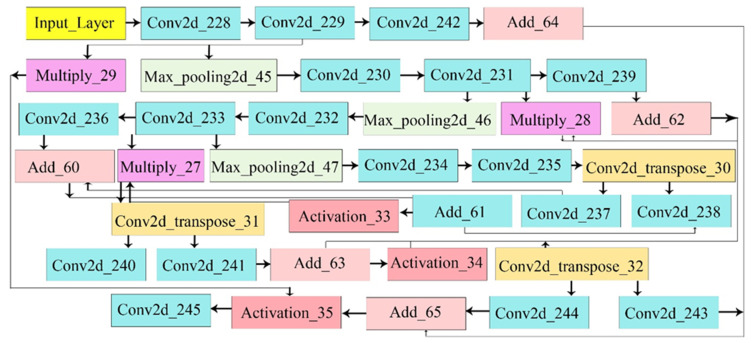
Proposed layer architecture of the segmentation model.

Table 2 presents the hyperparameters for segmentation, including a batch size of 8, a segmenter of U-Net, and 50 epochs, which yield excellent results. The layer descriptions of the U-Net technique with activations are stated in Figure 3.

## 4. Experimentation and Discussion

This study proposes a method for classifying stomach ulcers based on Q-learning with a CNN. This experiment is designed to differentiate between different classes of stomach ulcers. The overall experiments are performed on Intel(R) Core(TM) i9-14900KF (3.20 GHz), 32.0 GB RAM, Nvidia 4060 Ti 16 GB Graphic Card with a Windows operating system.

The approach is structured around two fundamental components: segmentation and classification. The proposed method’s performance will be evaluated on the three publicly available benchmark datasets and one local dataset, including Kvasir [37], Nerthus [38], CVC-ClinicDB [39], and the POF Private Dataset. The Kvasir includes 8000 records with eight classes, including Z-Line, Esophagitis, Cecum, Pylorus, Polyps, Ulcerative Colitis, Dyed and Lifted, Dyed Resection, and Margins. The CVC-ClinicDB dataset comprises 612 images and 612 corresponding masks, all of which belong to a single class: Polyps. The Nerthus dataset contains 5525 images with four classes: Inadequate, Borderline, Satisfactory, and Excellent. The POF Private Dataset contains 600 images with two classes such as Hemorrhoids and barretts. Data augmentation was applied using vertical and horizontal flips to balance the dataset and increase the number of images. The model was then trained and tested on a combined set of original and augmented images. After augmentation, the total number of original and augmented images for Kvasir, Nerthus, CVC-ClinicDB, Kvasir Segmentation, Nerthus Segmentation, and Private datasets is 16,000, 11,050, 2448, 3600, 1652, and 1200, respectively. The 0.5 hold-out validation is used, where half the data is used for training and half for testing. Table 3 presents the details of open-access publicly available datasets.

### 4.1. Experiment #1: Classification of Stomach Disease

The Q-learning algorithm, integrated with a CNN model, is employed to classify stomach ulcers. The classification is based on four categories defined in the Nerthus dataset: A-0, B-1, C-2, and D-3. Table 4 shows model training performance across 40 training episodes.

In Table 4, each episode represents a single training cycle, and the corresponding reward indicates the model’s performance during that cycle. Higher rewards generally reflect better model accuracy or decision-making. In the early episodes (1–10), the rewards range from 74 to 94, indicating some variation as the model begins to learn. This fluctuation is common during initial training when the model is adjusting its parameters. Episodes 1 and 3 demonstrate high performance, achieving rewards of 94 and 92, respectively, while Episode 10 yields a lower reward of 74. In the middle episodes (11 to 30), the rewards remain relatively stable, mostly between 82 and 92. Episode 28 records the highest reward of 96, indicating a peak in model performance. There are a few dips, such as Episodes 27 and 29, which have rewards of 78, but overall, this phase demonstrates consistent learning progress. The final episodes (31–40) continue this trend of stability. Rewards in this phase are tightly grouped between 80 and 90, suggesting that the model has reached a plateau in learning. This consistent performance indicates that the model has likely converged and is no longer making significant improvements, but is performing reliably. The testing classification results on the Nerthus Dataset are given in Table 5.

**Figure 4:**
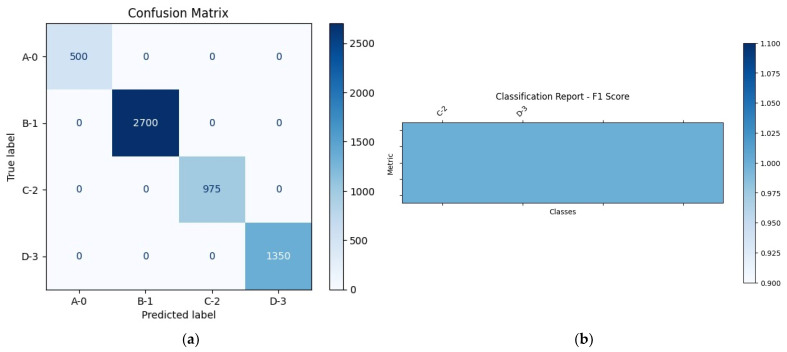
Performance evaluation of the proposed model on the Nerthus dataset: (**a**) confusion matrix and (**b**) classification report.

In Table 5, the Nerthus dataset model achieved perfect scores in all evaluation metrics for each class. The error rate on this dataset is 0, and the model achieves 100% reward in the testing phase. The results show that the model is highly effective for the given dataset and may be particularly well-suited to the structure and features of the Nerthus dataset. Figure 4 presents the performance evaluation of the proposed model on the Nerthus dataset.

**Figure 5:**
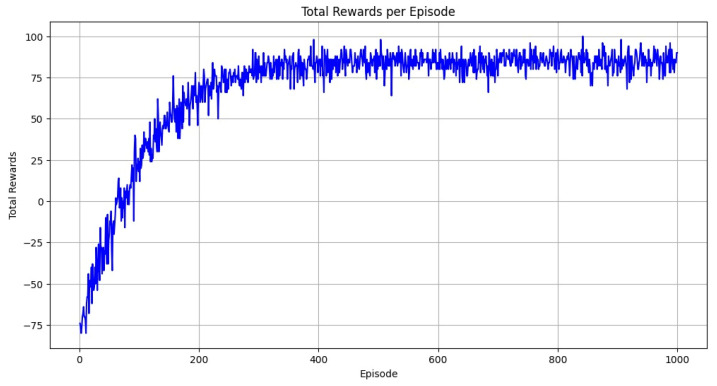
Represents total rewards on the Nerthus dataset.

In Figure 4a, the matrix reveals perfect classification, as all the values lie on the main diagonal. Specifically, the model accurately predicted 500 instances of A-0, 2700 instances of B-1, 975 instances of C-2, and 1350 instances of D-3, with no misclassifications or confusion between classes. This diagonal dominance confirms that the model achieved 100% accuracy in identifying each class. Figure 4b visualizes the classification report using an F1 Score chart. The uniform height and shading of the bars across all classes indicate consistent and perfect performance. The chart emphasizes that the model treated each class equally well, with no variation in classification quality. Together, these two figures demonstrate that the proposed model performed flawlessly on the Nerthus dataset, with complete accuracy and ideal precision–recall. This suggests either highly distinguishable data features within the dataset or a model exceptionally well-suited to the task. The model’s performance in terms of reward is visualized in Figure 5.

The Nerthus dataset achieves 100% performance across all metrics, thanks to the strong feature representation provided by the CNN and Q-learning optimization. Strict data partitioning and repeated validation confirmed that no feature or data leakage occurred. The testing classification results of the proposed model on the Kvasir dataset are shown in Table 6.

**Figure 6:**
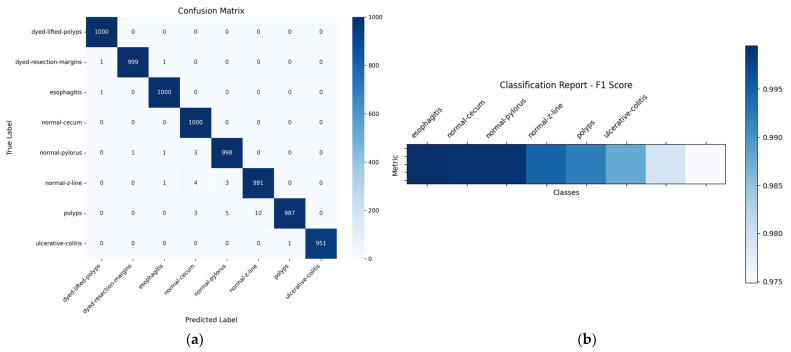
Performance evaluation of the proposed model on the Kvasir dataset: (**a**) confusion matrix and (**b**) classification report.

Table 6 presents the classification performance of the proposed model on the Kvasir dataset, which encompasses various gastrointestinal classes. Each row corresponds to a specific class, and the performance is evaluated using four key metrics: Accuracy, F1 Score, Recall, and Precision. For the classes DLP, DRM, and E, the model achieved perfect scores of 100% in F1 Score, Recall, and Precision, indicating flawless identification and classification without false positives or false negatives. Classes like Normal-cecum and Normal-pylorus also achieved nearly perfect scores, with F1 Scores and Precision at 99% and Recall at 100%, reflecting excellent generalization in detecting normal regions of the gastrointestinal tract. The Normal-z-line class shows slightly lower precision at 98%, although it still maintains high performance in other metrics, suggesting rare instances of false positives. For Polyps, the model attained a high F1 Score of 98%, Recall of 99%, and Precision of 97%. This indicates the model is very good at detecting polyps, though a small number may be misclassified. Ulcerative colitis has the lowest F1 Score (97%) and Recall (95%) among all classes, although it maintains a perfect Precision of 100%. This means that while the model is very accurate when it predicts ulcerative colitis, it may occasionally miss some true cases (lower Recall). Overall, the proposed model demonstrates high reliability and strong performance in classifying various gastrointestinal conditions and structures from the Kvasir dataset, with minor limitations in Recall for ulcerative colitis. Figure 6 shows the results on the Kvasir dataset.

**Figure 7:**
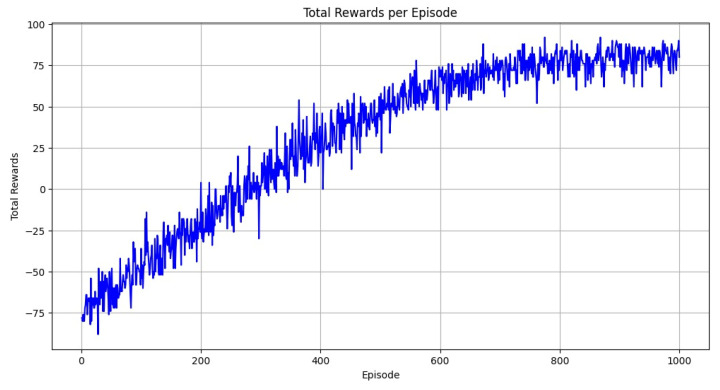
Visualization of the total rewards on the Kvasir dataset.

In Figure 6a, most of the values are concentrated along the diagonal, which means that the model is making accurate predictions for the majority of the classes. For instance, classes like “dyed-lifted-polyps,” “esophagitis,” and “normal-cecum” have almost perfect predictions, with very few or no mistakes. There are some misclassifications, particularly with the “ulcerative-colitis” class, where a few samples are incorrectly predicted as other similar classes like “polyps.” Even so, the number of incorrect predictions is very low overall, suggesting that the model is doing a great job at distinguishing between the different types. Figure 6b presents a classification report using F1 scores for each class, shown as a heatmap. The darker the color, the better the score. Most classes have very high F1 scores, close to 1.0, which means the model is both precise and consistent in its predictions. The class “ulcerative-colitis” again has the lowest score among them, but it is still quite high, indicating that the model performs well even on the harder cases. Figure 7 provides a visual summary of the reinforcement learning performance using the Kvasir dataset.

Table 7 compares the proposed novel classification model’s results with those of the existing method on different datasets.

The multiple models, including LBP, InceptionNet, ResNet50, VGG-16, PCA, and mRMR, are proposed for SU classification and achieved 95% accuracy on the KVASIR, NERTHUS, and stomach ulcer datasets [40]. The InceptionNetV3, GITNet, ACO, and AGCWD are proposed for classifying multiple classes of stomach ulcers using the NERTHUS dataset and achieve 99% accuracy [41]. Proposed models, such as BDA, ESKK, GA, and PSO, are utilized for classifying stomach ulcers using the NERTHUS dataset with an accuracy of 98.2% [15]. The VGG-19 and ResNet-50 models are utilized to detect stomach ulcers on the Kvasir dataset, achieving 96.8% accuracy [42]. RestNet50* is used to classify stomach ulcers on the Kvasir dataset [16]. Spatial-attention ConvMixer models are used for detection and classification using an SVM classifier. The model achieves 93.3% accuracy on the Kvasir and datasets [17]. The VGG19, ResNet50V2, ResNet152V2, EfficientNetV2B0, EfficientNetV2B3, InceptionV3, DenseNet201, and Xception models handle the imbalance class problem and classify stomach ulcers using the Kvasir dataset with an 88.6% accuracy [18]. A Vision Transformer combined with a CNN was used to achieve improved classification results on the Kvasir dataset [20].

The proposed model employs well-established concepts such as CNNs, Q-learning, and Attention U-Net, the originality of our work lies in the innovative integration and synergistic interaction of these components within a unified two-phase architecture designed specifically for gastrointestinal ulcer analysis. Existing studies, such as the SAC model [18] and Vision Transformer-based frameworks [20], have focused on improving either classification accuracy or feature representation within a single-task setup. Similarly, deep CNN-based approaches like VGG19 and ResNet variants [19] primarily rely on transfer learning to enhance classification but lack a mechanism for adaptive decision-making or lesion-focused segmentation refinement. In contrast, our proposed Q-learning-enhanced CNN introduces a reinforcement-driven optimization process, where a Q-table maps extracted feature states to optimal classification actions through continuous reward feedback, resulting in a dynamically adaptive and context-aware classifier.

### 4.2. Experiment #2: Segmentation of the Stomach Ulcer

**Figure 8:**
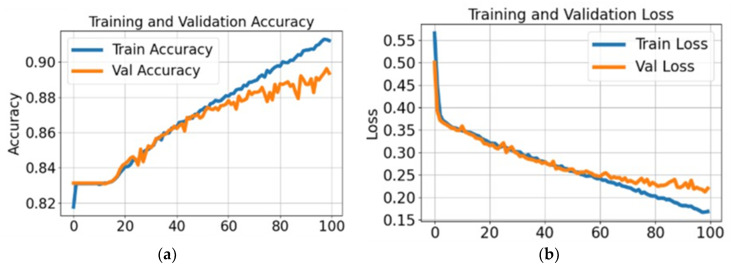
Training performance on the Kvasir dataset: (**a**) accuracy and (**b**) loss.

The performance of the proposed segmentation model has been evaluated using multiple measures, including Dice score, global accuracy (G-Accuracy), and weighted Intersection over Union (IoU). Figure 8 illustrates the training progression for the segmentation model using the Kvasir dataset.

In Figure 8, these results imply that the model is learning effectively and demonstrates strong generalization capability to unseen data, with only a minimal difference between training and validation performance. Figure 8b presents both training and validation loss during the same period. The blue line representing training loss shows a downward trend as the model learns. The validation loss, indicated in orange, follows the same trend and stabilizes after the initial drop. Together, these plots suggest that the segmentation model is effectively trained and demonstrates consistent performance on the Kvasir dataset. The proposed segmentation model results are computed using four different datasets: Kvasir, Nerthus, CVC-ClinicDB, and the POF Private dataset, as shown in Table 8.

Accuracy, Dice Score, and IoU are widely used to assess the performance of segmentation models. On the Kvasir dataset, the model performed very well, achieving 98.09% accuracy, 93.59% Dice Score, and 87.95% IoU. This means it correctly classifies and segments the region of interest with high precision. With the Nerthus dataset, the model showed even better results, reaching 99.77% accuracy, 98.71% Dice Score, and 97.46% IoU. These high scores indicate that the model had a profound understanding of the data and was able to produce highly accurate segmentations. The model, when evaluated on the CVC-ClinicDB dataset, also did quite well, with 98.49% accuracy, 91.89% Dice Score, and 85.00% IoU. The slightly lower scores compared to Nerthus may be due to differences in image quality or more challenging examples in this dataset; however, overall, the performance remains very strong. For the POF Private dataset, the model achieved 99.13% accuracy, which is high; however, the Dice Score and IoU were noticeably lower at 88.45% and 79.29%, respectively.

### 4.3. Visual Representation of Segmentation Dataset

**Figure 9:**
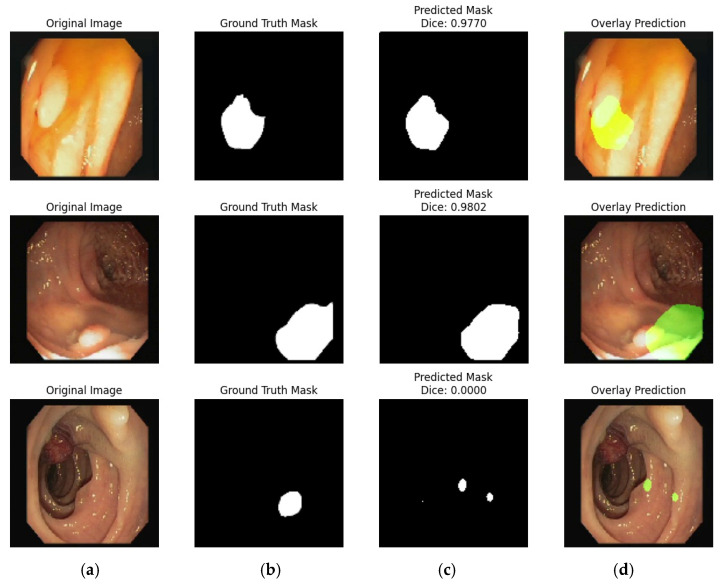
Illustration of segmentation on the Kvasir Dataset: (**a**) Original Image; (**b**) Ground Truth; (**c**) Model Prediction; and (**d**) Overlay Prediction.

The segmentation results on the Kvasir dataset are shown in Figure 9. Where each row represents a single test case and contains three images: (a) the original input image, (b) the corresponding ground truth mask created by experts, and (c) the predicted mask generated by the model, along with the Dice Similarity Coefficient (DSC) value for each prediction. The Dice scores for these examples are 0.9627, 0.9883, and 0.8787. The elevated DSC scores indicate that the model is performing well, exhibiting a small discrepancy between the predicted and actual segmented regions.

**Figure 10:**
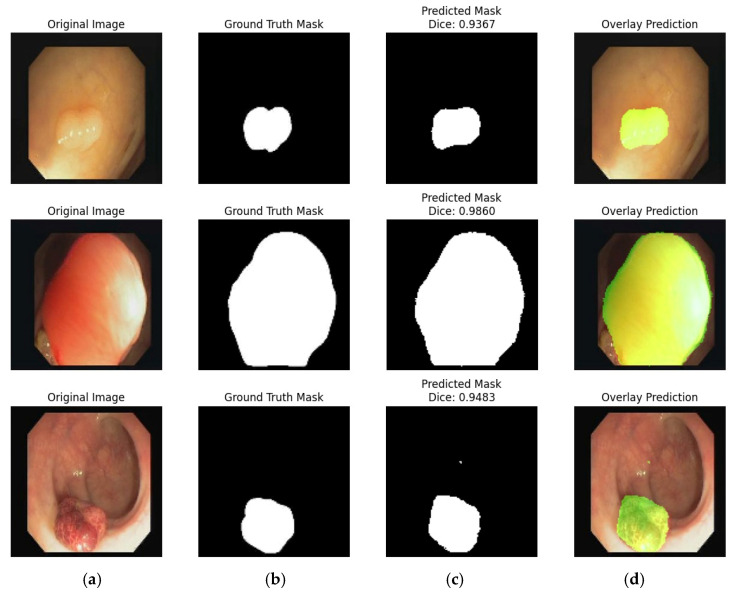
Segmentation result of SU: (**a**) Original Image; (**b**) Ground Truth Mask; (**c**) Predicted Mask; and (**d**) Overlay Prediction with Boundary.

Overall, Figure 9 visually confirms the strong segmentation performance of the model in the Kvasir dataset, with results that align well with the expert annotations. Figure 10 presents visual results of SU segmentation using the Nerthus dataset.

In Figure 10, each row illustrates a separate example of the model’s output, with four columns as shown in (a) original input image, (b) expert-annotated ground truth mask, (c) model-generated predicted mask, and (d) overlay of the prediction on the original image, where the segmented area is highlighted in green for clearer visual understanding. The Dice Similarity Coefficient (DSC) values in this figure are very high, at 0.9871, 0.9887, and 0.9897, indicating a strong alignment between the predicted masks and the ground truth. These values demonstrate that the model is highly accurate in identifying the ulcer regions. Looking at the overlay column (d), we can see that the model’s predicted boundaries align very closely with the actual ulcer regions in the input images. The green overlay helps to clearly visualize how well the model has segmented the affected areas. Overall, Figure 10 highlights the model’s strong performance in segmentation tasks on the Nerthus set. The predicted masks are almost identical to the ground truth, confirming that the model can reliably detect and segment stomach ulcers with high precision in these images. The proposed segmentation model’s results are compared with those of existing work and are depicted in Table 9.

On the Kvasir dataset, the existing techniques provide 91% accuracy using U-Net and double U-Net models [48]. DUCK-Net was used to segment the stomach ulcer on Kvasir and CVC-ClinicDB and achieved 98% accuracy [49]. The Li-SegPNet model is passed through the encoder and decoder modes for accurate segmentation on the Kavasir dataset [50]. Wavelet transformation and AdaptUNet (a Hybrid U-Net) are proposed for stomach ulcer segmentation, achieving 98% accuracy on the Kavasir dataset [43]. The DeeplabV3 segmentation model, combined with ResNet-50, is utilized to segment stomach ulcers using the Nerthus dataset, achieving an accuracy of 90% [51]. For the automatic segmentation of SU, a proposed encoder–decoder deep CNN model was used, and the model achieved 93% accuracy on the Nerthus datasets [52]. FEES and SimDCL achieved 73% and 81% accuracy, respectively, on the Nerthus and Kvasir datasets [45]. A fully convolutional neural network is applied for segmentation and evaluated on CVC-ClinicDB with 96% accuracy [53]. For the segmentation of SU, the doubleU-Net model was proposed, achieving 92% accuracy on the CVC-ClinicDB dataset [54]. An end-to-end fully CNN was proposed for the accurate segmentation of SU on the CVC-ClinicDB dataset and achieved 95% accuracy [47].

This research proposed a U-Net model for accurately segmenting the ulcer and achieved an accuracy of 98% and 99%. 99% and 79% on benchmark datasets Kvasir, Nerthus, CVC-ClinicDB, and the POF local dataset, respectively. This novel model is more effective compared to existing models. However, the proposed methodology has a limitation in that the model’s performance heavily depends on the quality and diversity of the training datasets, which may limit its generalizability to other GI diseases. Moreover, integrating classification and segmentation increases system complexity, making the deployment of real-time clinical systems challenging.

## 5. Conclusions

Ulcer detection in gastrointestinal imaging is a complex task due to the variable appearance of lesions, uneven illumination, and similarity with non-diseased regions. Manual methods are inefficient and prone to error, which necessitates the use of advanced automated techniques. Therefore, a deep reinforcement learning-based classification model is proposed. The model is built on the selected layers of a CNN model with Q-learning parameters that are trained from scratch using the optimum hyperparameters. The performance of the model is evaluated on two publicly available datasets, Nerthus and Kvasir. The proposed model provides 100% and 99.08% accuracy, respectively. The attention-based U-Net model is developed for segmenting classified images. The model’s performance is assessed on four datasets, including Kvasir, CVC-ClinicDB, Nerthus, and a private POF dataset, and achieves segmentation accuracies of 98.09%, 98.49%, 99.77%, and 99.13%, respectively. Corresponding Dice scores were 93.59% (Kvasir), 91.89% (CVC-ClinicDB), 98.71% (Nerthus), and 88.45% (POF), while the Intersection over Union (IoU) scores were 87.95%, 85.00%, 97.46%, and 79.29%. The obtained outcomes are superior to those in existing published works.

Future research directions include the integration of advanced enhancement techniques to improve the generalization of the model, such as using generative adversarial networks (GANs) to synthesize diverse training data, extending the framework to process and analyze multimodal gastrointestinal images to enhance segmentation accuracy further, optimizing the proposed method for real-time applications in clinical settings to ensure faster processing while maintaining accuracy, and expanding the scope of the proposed method to include automated stomach growth tracking and prognosis analysis to facilitate early interventions and personalized treatment. Addressing these areas, the proposed methodology can be further refined and expanded to effectively contribute to the diagnosis and treatment of stomach ulcers.

## Figures and Tables

**Table 1:** Hyperparameters of the proposed classification model.

Hyperparmeter	Value
α	0.1
γ	0.6
ε	0.1
Episodes	1000
Optimizer	AdamW
Batch Size	32
Img Size	(64, 64)
Num Classes	8
Loss	BinaryCrossentropy

**Table 2:** Hyperparameters of the Proposed Segmentation Model.

Hyperparameter	Value
Batch size	8
Segmenter	Attention-Based U-Net
Epochs	50

**Table 3:** Publicly available SU datasets.

Ref. #	Year	Datasets	Classes	Images
[37]	2017	Kvasir	8	8000
[38]	2017	Nerthus	4	5525
[39]	2015	CVC-ClinicDB	1	1224
[37]	2017	Kvasir Segmentation	1	1800
[38]	2017	Nerthus Segmentation	1	826
	2022	POF Private Dataset	2	600

**Table 4:** Proposed model training performance in terms of rewards and episodes.

Episodes	Rewards	Episodes	Rewards
1	94	21	94
2	84	22	84
3	92	23	88
4	90	24	84
5	86	25	88
6	82	26	92
7	92	27	78
8	88	28	96
9	86	29	78
10	74	30	92
11	82	31	92
12	88	32	84
13	84	33	80
14	84	34	86
15	82	35	78
16	74	36	86
17	90	37	84
18	82	38	84
19	76	39	90
20	76	40	90

**Table 5:** Proposed Classification Result on Nerthus Dataset.

Classes	Accuracy	F1 Score	Recall	Precision
A-0	100%	100%	100%	100%
B-1	100%	100%	100%	100%
C-2	100%	100%	100%	100%
D-3	100%	100%	100%	100%

**Table 6:** Proposed Classification Result on Kvasir Dataset.

Classes	Accuracy	F1 Score	Recall	Precision
Dyed-lifted-polyps (DLP)	99%	100%	100%	100%
Dyed-resection-margins (DRM)	99%	100%	100%	100%
Esophagitis (E)	99%	100%	100%	100%
Normal-cecum (NC)	99%	99%	100%	99%
Normal-pylorus (NP)	99%	99%	100%	99%
Normal-z-line (NZL)	99%	99%	99%	98%
Polyps (P)	99%	98%	99%	97%
Ulcerative-colitis (UC)	99%	97%	95%	100%

**Table 7:** Classification result compared with existing work.

Ref. #	Year	Datasets	Accuracy%
[40]	2021	Nerthus	95.0
[41]	2023	Kvasir	99.0
[15]	2024	Kvasir	98.2
[42]	2023	Kvasir	96.8
[16]	2024	Kvasir	92.0
[17]	2024	Kvasir	93.3
[18]	2024	Kvasir	88.6
[19]	2025	Kvasir	94.3 F-score
Proposed Method		Nerthus	100
		Kvasir	99.08

**Table 8:** Performance of the Segmentation Model using four Datasets.

Datasets	Accuracy	Dice Score	IOU	Epochs
Kvasir	0.9809	0.9359	0.8795	50
Nerthus	0.9977	0.9871	0.9746	50
CVC-ClinicDB	0.9849	0.9189	0.8500	50
POF Private dataset	0.9913	0.8845	0.7929	50

**Table 9:** Results comparison with existing methods on the same benchmark datasets.

Ref. #	Year	Datasets	Accuracy
[43]	2024	Kvasir	0.988
[44]	2025	Kvasir	0.897
[45]	2024	Nerthus	0.727
[46]	2024	CVC-ClinicDB	0.983
[47]	2025	CVC-ClinicDB	0.959
Proposed Method		Kvasir	0.980
		Nerthus	0.997
		CVC-ClinicDB	0.984
		POF private data	0.991

## Data Availability

The raw data supporting the conclusions of this article will be made available by the authors on request.
